# Child maltreatment associates with violent victimization in young adulthood: a Brazilian birth cohort study

**DOI:** 10.1186/s12889-023-17245-8

**Published:** 2023-11-20

**Authors:** Eveline Bordignon, Vanessa Iribarrem Avena Miranda, Christian Loret de Mola Zanatti, Ana Maria Baptista Menezes, Helen Denise Gonçalves da Silva, Fernando César Wehrmeister, Joseph Murray

**Affiliations:** 1 Postgraduate Program in Epidemiology, Department of Social Medicine, Federal University of Pelotas, Pelotas, RS Brazil; 2 Human Development and Violence Research Centre (DOVE), Federal University of Pelotas, Pelotas, Brazil; 3 Postgraduate Program in Public Health, Universidade do Extremo Sul Catarinense (UNESC), Criciúma, SC Brazil; 4 Universidade Federal do Rio Grande (FURG), Rio Grande, RS Brazil

**Keywords:** Maltreatment, Violence, Childhood, Young adulthood, Cohort

## Abstract

**Background:**

Maltreatment in childhood may leave people vulnerable to further experiences of violence and more severe effects of stress later in life. Longitudinal studies of risk for violent victimisation after maltreatment are lacking in low- and middle-income countries. The objective of this study was to quantify the risk for violent victimization in the family and community in young adulthood following experiences of childhood maltreatment (experiences of physical, emotional and sexual abuse and neglect) up to age 15 years in an urban Brazilian population.

**Methods:**

3246 participants in a prospective, population-based birth cohort study in Pelotas, Rio Grande do Sul, Brazil, were assessed at birth, 15 and 22 years. Sociodemographic factors were reported by mothers at birth and adolescents at age 15 years. Maltreatment and violent victimisation were self-reported in confidential questionnaires at 15 and 22, respectively. Multinomial logistic regression analyses estimated the association between having experienced any maltreatment and later experiences of family and community violence in young adulthood (no adult violence, violence only in the family context, only in the community, or both violence in the family and community), adjusting for sociodemographic factors.

**Results:**

39% of females and 27% of males reported any maltreatment up to age 15 years. At 22 years, rates of past year violence in the family or community were 17.6% for females and 20.2% for males. Maltreatment was strongly associated with community violence (Females: OR = 2.96, CI = 1.83–4.80; Males: OR = 2.01, 95%CI = 1.01-4.00) and its co-occurrence with family violence (Females: OR = 2.33, 95%CI = 1.34–4.04; Males: OR = 3.20, 95%CI = 1.82–5.65) in young adulthood, after adjustment for background sociodemographic factors.

**Conclusion:**

Childhood maltreatment is an important risk factor for later violent victimisation in both the family and community context. The effects of repeated trauma through the life-course needs research and clinical attention.

**Supplementary Information:**

The online version contains supplementary material available at 10.1186/s12889-023-17245-8.

## Introduction

The long-lasting health consequences of child maltreatment (any experience of physical, emotional, or sexual abuse, or neglect), as well as adverse socioeconomic outcomes in adulthood, have been well-documented [1–5]. Both biological and psychological mechanisms have been implicated in the adverse effects of neglect and abuse, particularly in relation to the stress response system, and the person’s sense of afety and trust in interpersonal relationships [6]. Recent research suggests that experience of early adversity can interact with later stressful experiences to produce even more toxic effects on health [7]. For example, in a nationwide study of adults in the USA, the impact of stressful events in the prior 12 months on mental disorders was about twice as large among individuals who had previously experienced multiple adversities in childhood compared to none [7]. However, evidence on the link between childhood maltreatment and later violence, a particularly severe form of stress, is limited and new studies are needed, particularly in low-and middle-income countries where child support services are under resourced and rates of violence are high [8].

Childhood maltreatment might predict risk for further violent victimisation, and thus continuity of stress across the life-course” of stress across the life-course, partly because of continuity in adverse environments conducive to victimisation [9]. Child maltreatment itself also might contribute to risk of future victimisation because of its impact on risk behaviours (such as alcohol or drug use, and aggressive behaviour) or because it predicts partnering, or interacting with more antisocial individuals [10, 11]. Later victimisation after maltreatment might occur in either the domestic or community context. In a large Canadian study, child maltreatment associated with increased risk for intimate partner violence, with larger effects for women than men [12]; however, like most studies of victimisation after child maltreatment, this survey had a cross-sectional design, and focused on only one type of violent outcome. Longitudinal studies are scarce and tend to include only women when examining risk of intimate partner violence (IPV) after child maltreatment. According to a systematic review in 2018 [13], only five longitudinal studies, all conducted in the United States, including a total of 1,516 women, had tested the association between childhood abuse and intimate partner violence against women, with a non-significant average effect (odds ratio = 1.3, 95%CI 0.93–1.80). Beyond IPV, there is a dearth of longitudinal evidence on how child maltreatment associates with family violence more generally in adulthood - committed by other family members as well as intimate partners.

Considering victimisation in the community, associations with prior child maltreatment may differ for women and men, particularly as males commit most community violence, and perpetration and victimisation are strongly associated [14].Although the “cycle of violence” literature has focused on the effects of maltreatment on the risk of *perpetrating* violence [15], less is known about risk for victimisation in the community. In the current study we present the epidemiology of maltreatment until age 15, and subsequent risk for experiences of violence in the family and community, in young adulthood at age 22 years, in a Brazilian longitudinal study. Prior evidence suggests that maltreatment tends to occur in an especially severe form in Brazil [16], that rates of violence against women are high [17], and serious community violence is an enormous social and public health problem [18]. Identifying links between these different forms of violence across the life-course is thus of great importance in this context.

## Methods

Data from the 1993 Pelotas Birth Cohort were used, which has a prospective longitudinal design. In 1993, all live births in the city’s maternity hospitals taking place between January 1 and December 31 were identified, and the mothers were invited to participate in the study. A total of 5249 individuals (response rate of 99.7%) were included, and they were followed from birth to 22 years of age. For analyses on maltreatment and later violent victimisation, information from the following follow-ups was used: perinatal visit (n = 5249), 15 years (n = 4349; 85.7% of eligible individuals were followed) and 22 years (n = 3810; 76.3% of eligible individuals were followed). At 22 years, REDcap software was used to collect data (10.1016/j.jbi.2008.08.010). Details about data collection are available in other published articles [19, 20]. See also the supplementary table.

### Measurements

#### Child maltreatment up to age 15 years

Experiences of child maltreatment were assessed at the 15-year follow-up through questions from the *Childhood Trauma Questionnaire* (CTQ) [21, 22]. Questions applied in this study were the following: (a) Have there ever been fights with physical aggression in your home between adults or has an adult ever assaulted a child or adolescent?; (b) Has it ever happened that you did not had enough food at home or that you put on dirty or torn clothes because you had no others?; (c) Have you ever thought or felt that your father or mother never wanted you to have been born?; (d) Have you ever thought or felt that someone in your family hates you?; (e) Has it ever happened that an adult in your family or someone who was taking care of you hit you in a way that left you hurt or with marks?; (f) Has anyone ever tried to do sexual things with you against your will, for which they threatened you or hurt you? (g) Have you ever been separated from your parents to be cared for by someone else? A dichotomous maltreatment variable was created by coding “1” if any answer to the above questions was yes, and coding “0” if no answer was given as yes. All questions refer temporally from the participants’ childhood to the time of the interview, i.e. when they were at the age of 15 years.

#### Victimisation at age 22 years

At the 22-year follow-up, family victimisation and community victimisation were measured in relation to the preceding one-year period. Family victimisation violence was recorded as aggression perpetrated by a family member (father, mother, brother, sister, uncle, aunt, companion, or other person) against the participant, and victimisation due to community violence was considered to consist of aggression perpetrated by someone who was not a family member. Questions about violent victimisation were taken from previous victimisation studies conducted in Brazil [23, 24] and were adapted to identify whether the act of violence was committed by a person within the family or in the community. These questions were then tested in a pilot study among male and female undergraduate students prior to application in the current study. The five questions, first asked about in relation to family members, and then in relation to people in the community were as follows: (a) How many times has someone made a serious threat to hurt you physically?; (b) How many times has someone hit you, pushed you, kicked you or physically assaulted you without a weapon?; (c) How many times has someone attacked you with a knife, firearm or other weapon?; (d) How many times has someone stolen an object from you, using violence or threats?; (e) How many times has someone grabbed, touched or assaulted your private sexual parts against your will? Affirmative answers (“yes”) to any of these questions were computed as a positive event regarding family victimisation (where the act had been perpetrated by a family member) and community victimisation (where the act had been perpetrated by a non-family member). A final 4-category variable was created regarding victimisation at age 22 years: no victimisation, victimisation in the family only, victimisation in the community only, and co-occurrence of family and community victimisation.

#### Sociodemographic characteristics

Sociodemographic characteristics of the participants are reported based on measures assessed in the perinatal period and at 15 years of age. The following variables were used from the perinatal period: sex of the child (male/female); self-reported maternal age, which was then categorized into age groups (< 20; 20 to 29; or 30 years or more); maternal education in complete years of study, categorized as 0–4, 5–8, 9–11 or 12 years or more; family income in minimum monthly wages, categorized into wealth quintiles as Q5 (richest), Q4, Q3, Q2 and Q1 (poorest); and maternal marital status (with partner/without partner). At the age of 15 years, self-reported skin colour was recorded: white, black, brown or others (indigenous and East Asian), considered in this study as a socially relevant variable, rather than a biological entity.

### Statistical analysis

First, the prevalence of young adult violent victimisation (considering separately: family violence only, community violence only, and both family and community violence) was presented according to sample sociodemographic characteristics and experience of maltreatment up to age 15 years. All these results are shown stratified by participant sex, because of different rates of exposure to violence for females and males, and because of potentially different consequences of maltreatment between by sex, as has been considered in the literature on mental health [25]. Associations between sociodemographic characteristics and violence outcomes were examined using Pearson’s chi-square test for dichotomous exposure variables and the heterogeneity test for categorical variables, and p < 0.05 was considered statistically significant.

In a second set of analyses, the association between maltreatment and young adult victimisation (considering separately: family violence only, community violence only, and both family and community violence) was examined in multinomial logistic regression (with no young adult victimisation as the reference category), adjusting for maternal and participant sociodemographic characteristics (used in continuous form for maternal age, family income, and maternal education). Adjusted odds ratios and 95% confidence intervals are reported, stratified by sex. One additional model using the whole sample was run to test for a possible interaction between sex and maltreatment in predicting later experiences of violence. The analyses were carried out in the Stata statistical software, version 15.1 (Stata Corporation, College Station, USA).

## Results

Tables 1 and 2 show the sociodemographic profile of females and males in the study, respectively, as well as experiences of maltreatment up to age 15, and violence experienced at age 22. Up to age 15 years, 39% (95%CI = 37.02; 41.46) of females and 27% (95%CI = 25.09; 29.49) of males reported maltreatment (p < 0.001 for sex difference). At 22 years, 17.5% (95%CI = 15.8; 19.4) of females reported violent victimisation in the previous year (8.7% in the family only, 5.3% in the community only, and 3.6% in both contexts), compared to 20.2% (95%CI = 18.3; 22.3) of males (12.8% in the family only, 3.0% in the community only, and 4.4% in both contexts); p = 0.054 for sex difference in the overall prevalence of violence at age 22.

**Table 1:** Maltreatment and young adult victimisation prevalence among females, according to sociodemographic factors in the 1993 Pelotas Birth cohort study

	Maltreatment up to Age 15			Violent Victimisation Age 22 Years			
	N	Any maltreatment %		N	Family Violence Only %	Community Violence Only %	Family and Community Violence %
**Maternal age (years)**		*p < 0.001*			*p = 0.573*		
< 20	304	45.7		294	8.8	5.4	2.0
20–29	993	40.8		907	9.3	4.7	3.9
≥ 30	567	33.0		504	7.5	6.2	4.0
**Maternal schooling (years)**		*p = 0.001*			*p = 0.158*		
0–4	509	46.4		461	6.5	6.3	5.0
5–8	869	37.9		784	9.1	4.8	3.1
9–11	338	34.6		314	8.3	4.8	3.5
≥ 12	145	32.4		143	14.0	5.6	2.1
**Mother with partner**		*p < 0.001*			*p = 0.495*		
Yes	1,654	37.8		1511	8.4	5.1	3.6
No	210	50.5		194	10.8	6.7	3.1
**Family income (quintiles)**		*p = 0.009*			*p = 0.133*		
Q1 (most poor)	351	43.0		316	6.6	4.7	5.3
Q2	397	41.3		374	7.2	5.6	2.7
Q3	332	43.1		297	10.4	5.1	6.1
Q4	377	32.9		341	10.0	5.0	2.1
Q5 (most rich)	370	35.4		345	9.6	5.8	2.6
**Skin Colour**		*p = 0.038*			*p = 0.012*		
White	1,170	36.8		1051	7.9	5.4	2.6
Black	285	41.1		235	13.2	3.4	6.0
Brown	343	44.9		289	9.0	5.5	5.2
Others*	66	43.9		50	2.0	4.0	4.0
**Maltreatment**					*p < 0.001*		
No	1,133			978	8.0	3.2	2.5
Yes	731			608	10.2	7.7	5.4
**Total**	**1,864**	**39.2**		**1,705**	**8.7**	**5.3**	**3.6**

Numbers are smaller for the violent victimisation analysis than maltreatment analysis given loss to follow-up at age 22

CI = confidence interval. P values refer to tests of whether maltreatment and violence were each equally distributed across variables shown in the lefthand column. * Others: Indigenous and East Asian

**Table 2:** Maltreatment and young adult victimisation prevalence among males, according to sociodemographic factors in the 1993 Pelotas Birth cohort study

	Maltreatment up to Age 15			Violent Victimisation Age 22 Years			
	N	Any maltreatment %		N	Family Violence Only %	Community Violence Only %	Family and Community Violence %
**Maternal age (years)**		*P = 0.17*			*p = 0.904*		
< 20	288	31.6		271	11.1	3.3	4.8
20–29	841	26.6		825	13.8	3.0	4.4
≥ 30	446	25.6		445	11.9	2.7	4.3
**Maternal schooling (years)**		*p = 0.143*			*p < 0.001*		
0–4	396	30.8		375	10.4	4.5	7.7
5–8	762	27.0		730	12.2	2.6	4.7
9–11	289	25.3		301	15.9	2.0	1.3
≥ 12	127	21.3		134	15.7	3.0	0.7
**Mother with partner**		*p = 0.002*			*p = 0.087*		
Yes	1,367	25,9		1,347	12.9	2.6	4.2
No	208	36,1		194	11.9	5.7	5.7
**Family income (quintiles)**		*p = 0.334*			*p < 0.001*		
Q1 (most poor)	283	29.3		283	11.7	3.9	10.3
Q2	376	27.4		360	11.4	2.2	3.6
Q3	266	30.5		249	12.0	2.8	2.4
Q4	316	24.7		316	14.2	2.8	4.1
Q5 (most rich)	307	24.1		307	14.3	2.9	1.6
**Skin Colour**		*p < 0.001*			*p = 0.003*		
White	1,016	24.2		933	12.9	2.1	3.1
Black	230	27.4		212	11.3	4.3	8.5
Brown	263	36.9		240	14.6	5.0	6.3
Others*	65	35.4		61	11.5	0.0	3.3
**Maltreatment**					*p < 0.001*		
No	1,146			1,008	13.0	2.1	2.7
Yes	429			380	13.4	4.2	7.6
**Total**	**1,575**	**27.2**		**1,541**	**12.8**	**3.0**	**4.4**

Numbers are smaller for the violent victimisation analysis given loss to follow-up at age 22

CI = confidence interval. P values refer to tests of whether maltreatment and violence were each equally distributed across variables shown in the lefthand column. * Others: Indigenous and East Asian

Maltreatment of females was more common among children with mothers who were young, less well educated, with lower family income, without a partner, and participants with brown or black skin colour (Table 1). Experiences of family and community violence in young adulthood were associated with skin colour (mostly higher rates for people of brown and black skin colour), and maltreatment by age 15 for females. For males, Table 2 shows that maltreatment was more common among children whose mothers did not have a partner, and participants with brown or other skin colour. Experiences of family and community violence in young adulthood were associated with maternal schooling, family income, skin colour, and maltreatment by age 15 for males. Experiencing *both* family and community violence at age 22 years was reported at a higher rate by youth with lower maternal schooling, lower family income, brown and black skin colour, and maltreatment experiences.

Table 3 shows the results of multinomial logistic regression, examining the association between maltreatment and young adult violent victimisation, adjusting for sociodemographic factors. For both females and males, maltreatment was associated with over twice the odds of experiencing both family and community violence in young adulthood. Associations between maltreatment and community victimisation only were also large for both females and males. However, associations between maltreatment and victimisation in the family were weak, and only significant for females. Across these three categories of young adult victimisation, there was no evidence that associations with earlier maltreatment significantly differed for females and males (all p values for interaction terms > 0.40).

**Table 3:** Unadjusted and adjusted associations between maltreatment and young adult victimisation, among (A) Females, and (B) Males

	Family Violence Only	Community Violence Only	Family and Community Violence
	OR (95%CI)	OR (95%CI)	OR (95%CI)
A. Maltreatment among females			
Unadjusted	1.44 (1.01, 2.05)	2.75 (1.72, 4.39)	2.49 (1.46. 4.27)
Adjusted*	1.46 (1.02, 2.10)	2.82 (1.75, 4.56)	2.44 (1.41, 4.21)
**B. Maltreatment among males**			
Unadjusted	1.14 (0.80, 1.61)	2.22 (1.14, 4.32)	3.14 (1.82, 5.39)
Adjusted*	1.17 (0.82, 1.67)	1.99 (1.00, 3.96)	3.05 (1.74, 5.35)

Reference category for young adult victimisation outcome variable is no violent victimisation at age 22 years

OR = odds ratio. CI = Confidence interval

*Adjusted for maternal age, family income, maternal education, maternal marital status, participant skin colour (N = 1,554 females; N = 1,363 males)

## Discussion

In a large population-based, prospective cohort study in Brazil, child maltreatment was strongly associated with later victimisation in both the family and community environments, and associations were similar for females and males. Although childhood socioeconomic conditions were associated with various forms of violence, childhood maltreatment predicted young adult victimisation in the family and community, independently of socioeconomic factors, demonstrating important continuity between child maltreatment and young adult victimisation independent of socioeconomic background.

Despite decades of studies demonstrating adverse biopsychosocial consequences of maltreatment [26], surprisingly little evidence is available on the longitudinal links between maltreatment and further victimisation, especially in the transition to adulthood. In the current study, experiencing maltreatment up to age 15 was associated with over double the odds of suffering community violence on its own, or both community violence and family violence in young adulthood. Although this increased vulnerability could reflect continuity in risky environments, the association remained adjusting for several important socioeconomic factors, suggesting that child maltreatment itself might also involve pathways of psychosocial and behavioural change, leading towards further victimisation.

Cascading experiences of victimisation through the life-course may put individuals at particular risk for poor health and social outcomes. According to the stress sensitisation hypothesis, individuals with stressful experiences such as maltreatment in early life may be particularly vulnerable to poor health when confronted by new experiences of stress later in the life-course [7]. As such, the risk of further victimisation after maltreatment in the current study points to experiences of multiple forms of stress through the life-course that are potentially particularly threatening to health, and may have clinical implications for people suffering violence across these multiple contexts [27].

In the current study, females were more exposed to any child maltreatment, as well as family violence in young adulthood, consistent with studies across many other populations [26, 28–30]. Violence against girls and women, especially in the family environment, is a major public health and human rights problem [31]. Worldwide, it is estimated 30% of women are exposed to lifetime violence perpetrated by intimate partners, or sexual abuse, although rates vary according to the country and the methodology applied [32]. A culture of machismo in Latin America is still a critical issue to address in preventing violence against women in this region, as well as empowering women, and reinforcing laws to combat violence against women [33]. Like in other studies [34–36], young men in this population were at greater risk of exposure to community victimisation, which is consistent with Brazilian official statistics showing that the highest rates of mortality caused by violence (homicides) are among young men aged 15–29 years [37]. While sex differences in health and social exposures across the life-course are deeply complex phenomena, relating to cultural, social and biological processes, the higher rate of exposure to community violence among young men may be understood partly in relation to a higher likelihood that young men are involved in risky activities in the community environment, including perpetration of crime and violence, as local epidemiological studies have reported [38, 39]. Gender studies also reveal processes of violence socialization among boys as they seek to confirm a masculine identity, leading them to become more frequently involved in risky situations leading to higher rates of community victimisation and early mortality, and that these issues are exacerbated by social inequalities in Brazil [40, 41].

Among the main strengths of this study is its large sample size based on a total population, its prospective design, and consideration of multiple contexts of violence, for both females and males. Among study limitations, the following should be considered. First, although maltreatment was measured at age 15, and many studies use retrospective reports taken later in adulthood [42]. The current study measure is still retrospective across childhood and may have been subject to memory bias, reducing reporting of maltreatment. Second, this study used an abbreviated set of questions on maltreatment that did not permit sub-analyses by type of maltreatment, and a questionnaire on violence in adulthood that was specific to the Brazilian population, which limits comparisons with other studies. In a large study, losses over a 22-year period of this study are inevitable, and may have biased the results. Those excluded from the analysis are similar to those included according to gender, maternal age and mother’s marital status and different according to maternal education, family income and skin color of the young person, compared to those included in the sample.

Among the limitations of the study, it is important to recognise that causal inference about the effects of maltreatment on later violence exposure was not the aim of the study, and cannot be made on the basis of the associations that were tested. In particular, absence of paternal data and data on other factors in the complex etiology of victimisation, such as the neighbourhood context were not included. Also, the socioeconomic conditions that were associated with violence in this study were examined in a purely descriptive form. We recognise that these socioeconomic factors are highly interdependent, and causal inference about any single factor is not intended. Another important limitation is that the measure of maltreatment at age 15 years is retrospective regarding childhood exposure and is subject to memory bias.

In conclusion, this study shows significant continuity in violent victimisation through the life-course, in a Brazilian population. Specifically, child maltreatment was strongly associated with later experiences of violent victimization in the family and in the community in young adulthood. Although health impacts of victimisation were not investigated in the current study, the association between maltreatment and later violence highlights clinical issues of concern. When maltreatment occurs, vulnerability towards further victimisation, not only in the immediate context, but in later life phases, requires attention. When violent victimisation occurs in early adult life, a history of maltreatment may be particularly significant to consider regarding concurrent traumatic responses.

### Electronic supplementary material

Below is the link to the electronic supplementary material.

Supplementary Material 1

## Data Availability

Questionnaires and other study details are available at http://www.epi.demio-ufpel.org.br/site/content/coorte_1993/index.php. For more information for proposed collaboration or to gain access to the 1993 cohort data, potential partners should contact the researchers and then download the ‘Form for projects that do not involve data collection’.

## Declarations

All methods were carried out in accordance with relevant guidelines and regulations or Declaration of Helsinki.

## References

[1] Gallo EAG, De Mola CL, Wehrmeister F, Gonçalves H, Kieling C, Murray J (2017). Childhood maltreatment preceding depressive disorder at age 18 years: a prospective Brazilian birth cohort study. J Affect Disord.

[2] Gibb BE, Alloy LB, Abramson LY, Rose DT, Whitehouse WG, Donovan P (2001). History of Childhood Maltreatment, negative cognitive styles, and episodes of Depression in Adulthood. Cogn Therapy Res.

[3] Leitzke BT, Pollak SD, Deater-Deckard K, Panneton R (2017). Child maltreatment: consequences, mechanisms, and implications for parenting. Parental stress and early child development: adaptive and maladaptive outcomes.

[4] Smith CA, Ireland TO, Thornberry TP (2005). Adolescent maltreatment and its impact on young adult antisocial behavior. Child Abuse Negl.

[5] Norman RE, Byambaa M, De R, Butchart A, Scott J, Vos T (2012). The Long-Term Health consequences of child physical abuse, emotional abuse, and neglect: a systematic review and Meta-analysis. PLoS Med.

[6] Nelson CA, Scott RD, Bhutta ZA, Harris NB, Danese A, Samara M (2020). Adversity in childhood is linked to mental and physical health throughout life. BMJ (Clinical Research ed).

[7] McLaughlin KA, Conron KJ, Koenen KC, Gilman SE (2010). Childhood adversity, adult stressful life events, and risk of past-year psychiatric disorder: a test of the stress sensitization hypothesis in a population-based sample of adults. Psychol Med.

[8] Montoya O, Bhate-Deosthali P, Kilonzo N, Watts C. The relationship between poverty and Child Abuse and neglect: New evidence. University of Huddersfield; 2022.

[9] Mendes DD, Mari JJ, Singer M, Barros GM, Mello AF. Estudo De revisão Dos fatores biológicos, sociais e ambientais associados com o comportamento agressivo. Brazilian J Psychiatry. 2009;31. 10.1590/s1516-44462009000600006 19967203

[10] Oliveira PAd, Scivoletto S, Cunha PJ. Estudos neuropsicológicos E de neuroimagem associados ao estresse emocional na infância e adolescência. Archives of Clinical Psychiatry (São Paulo). 2010;37.

[11] Fava DC, Pacheco JTB (2017). Maus tratos, problemas de comportamento e autoestima em adolescentes. Revista Brasileira De Terapias Cognitivas.

[12] Shields M, Tonmyr L, Hovdestad WE, Gonzalez A, MacMillan H (2020). Exposure to family Violence from childhood to adulthood. BMC Public Health.

[13] Yakubovich AR, Stöckl H, Murray J, Melendez-Torres GJ, Steinert JI, Glavin CEY (2018). Risk and Protective Factors for Intimate Partner Violence Against Women: systematic review and Meta-analyses of prospective–longitudinal studies. Am J Public Health.

[14] Lauritsen JL, Laub JH (2007). Understanding the link between victimization and offending: New reflections on an old idea. Crime Prev Stud.

[15] Widom CS (1989). The cycle of Violence. Science.

[16] Viola TW, Salum GA, Kluwe-Schiavon B, Sanvicente-Vieira B, Levandowski ML, Grassi-Oliveira R (2016). The influence of geographical and economic factors in estimates of childhood abuse and neglect using the Childhood Trauma Questionnaire: a worldwide meta-regression analysis. Child Abuse Negl.

[17] Schraiber LB, D’Oliveira AFPL, França-Junior I, Diniz S, Portella AP, Ludermir AB (2007). Prevalência Da violência contra a mulher por parceiro íntimo em regiões do Brasil. Revista De Saúde Pública.

[18] Murray J, Cerqueira DRC, Kahn T (2013). Crime and Violence in Brazil: systematic review of time trends, prevalence rates and risk factors. Aggress Violent Beh.

[19] Goncalves H, Assuncao MC, Wehrmeister FC, Oliveira IO, Barros FC, Victora CG (2014). Cohort profile update: the 1993 Pelotas (Brazil) birth cohort follow-up visits in adolescence. Int J Epidemiol.

[20] Harris PATR, Thielke R, Payne J, Gonzalez N, Conde JG (2009). Esearch electronic data capture (REDCap)—A metadata-driven methodology and workflow process for providing translational research informatics support. J Biomed Inform.

[21] Grassi-Oliveira RSL, Pezzi JC (2006). Tradução E validação De conteúdo Da versão em português do Childhood Trauma Questionnaire. Revista De Saúde Pública.

[22] Humphreys KLLJ, Wear JG, Piersiak HA, Lee A, Gotlib IH (2020). Child maltreatment and depression: a meta-analysis of studies using the Childhood Trauma Questionnaire. Child Abuse Negl.

[23] Braga LLDAD (2012). Exposição à violência em adolescentes de diferentes contextos: família e instituições. Estudos De Psicologia.

[24] IdP D. Pesquisa Nacional De Vitimização. Universidade Federal de Minas Gerais; 2013.

[25] Gallo EAG, Munhoz TN, Loret de Mola C, Murray J (2018). Gender differences in the effects of childhood maltreatment on adult depression and anxiety: a systematic review and meta-analysis. Child Abuse Negl.

[26] Aho N, Gren-Landell M, Svedin CG (2016). The prevalence of potentially victimizing events, Poly-Victimization, and its Association to Sociodemographic Factors: a Swedish Youth Survey. J Interpers Violence.

[27] Lupien SJ, McEwen BS, Gunnar MR, Heim C (2009). Effects of stress throughout the lifespan on the brain, behaviour and cognition. Nat Rev Neurosci.

[28] Soler L, Paretilla C, Kirchner T, Forns M (2012). Effects of poly-victimization on self-esteem and post-traumatic stress symptoms in Spanish adolescents. Eur Child Adolesc Psychiatry.

[29] Gallo EAGDMC, Wehrmeister F, Gonçalves H, Kieling C, Murray J (2017). Childhood maltreatment preceding depressive disorder at age 18 years: a prospective Brazilian birth cohort study. J Affect Disord.

[30] Mossige S, Huang L (2017). Poly-victimization in a Norwegian adolescent population: prevalence, social and psychological profile, and detrimental effects. PLoS ONE.

[31] García-Moreno C, Zimmerman C, Morris-Gehring A, Heise L, Amin A, Abrahams N (2014). Addressing Violence against women: a call to action. The Lancet.

[32] World Health Organization (2013). Global and regional estimates of Violence against women: prevalence and health effects of intimate partner Violence and non-partner sexual Violence.

[33] Gherardi N (2016). Violência contra mulheres na América Latina. SUR - Revista Internacional De Direitos Humanos.

[34] Indias García S (2017). dPOJ. Lifetime victimization among Spanish adolescents. Psicothema.

[35] Sui XMK, Kessels LTE, Reddy PS, Ruiter RAC, Sanders-Phillips K. Violence exposure in South African adolescents: Differential and Cumulative effects on psychological functioning. J Interpers Violence. 2018. 10.1177/0886260518788363 PMC8064538 30024299

[36] Wright EMFA, Pinchevsky GM (2013). The effects of exposure to Violence and victimization across life domains on adolescent substance use. Child Abuse Negl.

[37] Cerqueira D, Ferreira H, Bueno S, Alves PP, Lima RSd, Marques D, et al. Atlas Da Violência 2021. Fórum Brasileiro de Segurança Pública; 2021.

[38] Murray J, Menezes AM, Hickman M, Maughan B, Gallo EA, Matijasevich A (2015). Childhood behaviour problems predict crime and Violence in late adolescence: Brazilian and British birth cohort studies. Soc Psychiatry Psychiatr Epidemiol.

[39] Bozzini AB, Maruyama JM, Santos IS, Murray J, Tovo-Rodrigues L, Munhoz TN (2023). Prevalence of adolescent risk behaviors at 11 and 15 years of age: data from the 2004 Pelotas birth cohort. Revista brasileira de psiquiatria (Sao Paulo Brazil: 1999).

[40] Schraiber LB, Barros CRS, Couto MT, Figueiredo WS. Albuquerque FPd. Homens, masculinidade e violência: estudo em serviços de atenção primária à saúde. Rev bras Epidemiol. 2012;15.

[41] Souza ERd (2005). Masculinidade E violência no Brasil: contribuições para a reflexão no campo da saúde. Ciência & saúde Coletiva.

[42] Hughes K, Bellis MA, Hardcastle KA, Sethi D, Butchart A, Mikton C (2017). The effect of multiple adverse childhood experiences on health: a systematic review and meta-analysis. Lancet Public Health.

