# Supplementary material for: Child maltreatment associates with violent victimization in young adulthood: a Brazilian birth cohort study
Source: BMC Public Health. 2023 Nov 20;23:2287. doi: 10.1186/s12889-023-17245-8 (PMC10658884; doi:10.1186/s12889-023-17245-8)
Supplement: Supplementary file 1 — Supplementary Material 1 [file 12889_2023_17245_MOESM1_ESM.docx]

**Supplementary Table.** Characteristics of participants in the 1993 Pelotas Birth Cohort Study included in the analyses, and participants not included

|  | **Included**  **in analyses** | **Not included**  **in analyses** | **p value** |
| --- | --- | --- | --- |
|  | **N (%)** | **N (%)** |  |
| **Child sex at birth** |  |  |  |
| Female | 1554 (53.3) | 475 (53.0) |  |
| Male | 1363 (46.7) | 422 (47.0) | 0.867 |
| **Maternal age (years)** |  |  |  |
| <20 anos | 488 (17.1) | 417 (17.9) | 0.667 |
| 20-29 | 1556 (53.3) | 1244 (53.4) |  |
| ≥30 | 863 (29.6) | 670 (28.7) |  |
| **Maternal schooling (years)** |  |  |  |
| 0-4 | 739 (25.3) | 729 (31.4) | <0.001 |
| 5-8 | 1381 (47.3) | 1043 (44.9) |  |
| 9-11 | 552 (18.9) | 371 (16.0) |  |
| ≥12 | 245 (8.4) | 182 (7.8) |  |
| **Mother with partner** |  |  |  |
| Yes | 2577 (88.3) | 2023 (86.7) | 0.081 |
| No | 340 (11.7) | 309 (13.2) |  |
| **Family income (quintiles)** |  |  |  |
| Q1 (most poor) | 538 (18.4) | 493 (22.2) | 0.005 |
| Q2 | 675 (23.1) | 520 (23.4) |  |
| Q3 | 505 (17.3) | 384 (17.3) |  |
| Q4 | 604 (20.7) | 397 (17.9) |  |
| Q5 (most rich) | 595 (20.4) | 426 (19.2) |  |
| **Skin Colour** |  |  |  |
| White | 1894 (64.9) | 875 (62.2) | 0.014 |
| Black | 426 (14.6) | 185 (13.2) |  |
| Brown | 492 (16.9) | 292 (20.8) |  |
| Others* | 105 (3.6) | 54 (3.8) |  |
| **Total** | **2,917 (55.6)** | **2,332 (44.4)** |  |

* Others: Indigenous and East Asian.
